# Exploring breast cancer stigma among medical students in Egypt: a national multi-center cross-sectional study

**DOI:** 10.1186/s12889-025-24656-2

**Published:** 2025-10-27

**Authors:** Mohamed Ahmed Zoromba, Hana Mohamed Deibes, Dareen H. Abdelhady, Walaa Mostafa Mokhtar, Abeer M. Kamal, Fatima Alhussein Jumma, Aya M. Eltantawy, Aalaa Abdelwahed Ahmed, Mazen Essam, Abdallah N. Alsaaidy, Salha Gourashi, Abdel-Hady El-Gilany, B. C. S. team of collaborators, Sameh Wahed, Mariam Mohamed Mansour, Malak S. Zidan, Salma Hamed, AbdelRahman Esmail, Selwan Emam, Habiba W. Hamed, Jana A. Merzban, NourAlhoda Mansour, Basel Tarabeh, Jana Samir, Dina Elatal, Nourelhoda Massoud, Ahmed B. Habib, Sohaila Khaled Yousef, Nada Shabaan Mohamed, Maryam Elshehawi, Salma T. Abdelrahman, Hazem Khaled, Radwa Yassin, Ahmed E. Elbanhawy, Mohamed Abdelhamed, Sohaila Faisal, Mohammed Kamal, Rageed K. Al Refa’I, Mahmoud Shehata, Mariam El-orabi, Ali M. Kamal, Basmala Mohamed, Asma Mohammed, Aseel Abbas, Sohaila Mohammed Al Refa’I, Saad Saber, Ibrahim Mohamed

**Affiliations:** 1 Faculty of Medicine, Mansoura University, Mansoura, Egypt; 2 Faculty of Medicine, Minia University, Minia, Egypt; 3 Faculty of Medicine, Alexandria University, Alexandria, Egypt; 4 Faculty of Medicine, Ain Shams University, Cairo, Egypt; 5 Public Health and Community Medicine Department, Faculty of Medicine, Mansoura University, Mansoura, Egypt

**Keywords:** Breast cancer stigma, Medical students, Egypt, Cancer stigma scale, Low- and middle-income countries

## Abstract

**Background:**

Breast cancer (BC) stigma remains an understudied barrier to early detection and quality care, particularly in low- and middle-income countries (LMICs). Yet, there is a lack of studies that assessed BC stigma among medical students worldwide; a critical gap given their future roles as healthcare providers. This study aims to explore BC stigma among medical students in Egypt and identify its associated factors.

**Methods:**

A national multi-center cross-sectional study was conducted during the academic Year 2024–2025, involving a convenience sample of 2,576 medical students from eight medical faculties across Egypt’s four main regions. Data were collected through an online questionnaire capturing socio-demographic characteristics, educational background, BC-related experiences, and knowledge of BC including students’ primary sources of information on the subject, alongside the BC-adapted Cancer Stigma Scale (CASS) that assessed six domains: Severity, Financial Discrimination, Personal Responsibility, Awkwardness, Avoidance, and Policy Opposition. Median (M) scores were computed, and non-parametric tests and multivariate linear regression were used to identify associated factors.

**Results:**

The overall BC stigma score was relatively low, with a median of 2.16 (36% of the total score). However, stigma was highest in Severity and Financial Discrimination (M = 3.0 and 2.67, respectively), and lowest in Avoidance and Policy Opposition (M = 1.00 and 1.33, respectively). Significantly higher stigma scores were observed in males (M = 2.20), those living in Upper Egypt governorates and rural residents (M = 2.30 and 2.24; respectively), and those with less-than-sufficient family income (M = 2.30). The regression analysis revealed significant associations between stigma and various sociodemographic variables, including sex, age, educational year, residence, and family income (*p*-values < 0.001), and BC-related questions such as history of breast problems and sufficient awareness about BC (both *p* < 0.001). The primary sources of information about BC for medical students were found to be medical curricula (24.2%), internet/social media (20.0%), and healthcare professionals (16.3%).

**Conclusion:**

This study provides an insight into exploring BC stigma among medical students. While the overall stigma was relatively low, significant disparities persist, particularly regarding perceived Severity among male and rural participants. Future interventions should target students with high levels of stigma through curricular reforms that incorporate anti-stigma training.

**Trial Registration:**

Not applicable.

**Supplementary Information:**

The online version contains supplementary material available at 10.1186/s12889-025-24656-2.

## Introduction

Globally, breast cancer (BC) is the second most diagnosed cancer and the leading cause of cancer-related deaths in women [1]. In Egypt, according to the latest Global Cancer Observatory (GLOBOCAN) report (2022), BC is the most common cancer among females, accounting for 34.9% of all cancer cases [2].

BC can be successfully treated if diagnosed early. However, this is not often the case in Egypt, where most cases present as either locally advanced or metastatic [3]. This reflects a broader pattern, common in low- and middle-income countries (LMICs), where limited healthcare resources and a predominant focus on curative rather than preventive care impede timely detection [4]. To reduce BC’s rising mortality, the Egyptian government launched Egypt's first national screening program, "Women Health Outreach Program" (WHOP) in 2007, and “Egyptian Women’s Health Initiative" (EWHI) in 2019 [5, 6]. Despite these efforts, women continue to face significant barriers in utilizing these initiatives, undermining their full potential. Reducing the number of late-stage BC cases in Egypt remains a critical public health priority, one that requires an in-depth understanding of the factors contributing to these delayed presentations. Existing research classifies these factors into three main categories: personal, economic, and healthcare system-related barriers. They include: a general tendency to delay health consultation (often due to financial constraints or fear of diagnosis), limited access to healthcare, lack of awareness, and – notably in Arab communities like Egypt – disease-related stigma [7–9].

Among these barriers, disease-related stigma stands out. Goffman, a foundational theorist in this area, first defined stigma as an attribute that associates a person with an unfavorable stereotype [10]. He claimed that in the eyes of others, the stigmatized individual is reduced from a whole and normal person to one who is shamed or disregarded [10]. "Disease-related stigma" is a term that refers to individuals facing discrimination, isolation, rejection, and/or criticism simply due to having suffered from a certain health condition [11]. Cancer-related stigma, in particular, exists due to a combination of certain myths, lack of understanding, and sociocultural beliefs, especially pronounced in Arab societies [9]. Among BC patients, stigma often arises from widespread misconceptions, such as fears that the disease is contagious or beliefs that it is a divine punishment from God for past sins. These perceptions can lead others to distance themselves from those with BC, prompting many women to blame themselves for their illness and conceal their symptoms to escape potential social isolation by others [9]. Such predicaments can dissuade women from seeking medical assistance, thereby delaying their diagnosis and treatment, and reducing their chances of survival [12].

On top of that, at a time when they are at their most vulnerable, patients still face stigma even in healthcare facilities [13–15]. They can be subjected to outright refusal of care, inadequate treatment, long waiting hours, verbal and physical abuse, or having their care assigned to less experienced personnel [15]. Patients place significant trust in healthcare providers and often rely on doctors to guide them through the proper clinical process, so much so that, in cases of BC, some may forgo screening unless explicitly advised by a doctor [7]. This highlights the critical role healthcare staff play in encouraging positive health-seeking behaviors and countering harmful attitudes. Therefore, not only is it essential that physicians convey accurate facts to both the public and patients, but also being more empathetic and impartial themselves can help shape an ideal patient experience, and encourage compliance, improving both the physical and mental health of patients [16, 17].

Addressing stigma towards BC patients early, particularly during medical school, is critical, as future doctors’ perceptions will directly influence patient care. While numerous studies assessed medical and non-medical students' knowledge, awareness, and attitudes toward BC, both internationally and in Egypt [18–20], those examining attitudes mostly focused on students’ perception of BC screening effectiveness and their willingness to undergo testing. However, no studies specifically investigated medical students’ stigma towards BC patients, leaving a critical gap that hinders the development of effective educational interventions to improve patients’ outcomes. This study aims to explore the key dimensions of BC–related stigma among medical students in Egypt and identify its associated factors, to inform future curriculum reforms and public health strategies to reduce stigma and improve outcomes in Egypt and beyond.

## Methodology

### Study design and setting

A national multi-center cross-sectional study with analytical and correlational components was conducted from January 2025 to May 2025. This study took place at the medical faculties of public universities across Egypt's four main geographical regions: Urban region (Alexandria University and Ain Shams University), Upper Egypt (Minia University and Fayoum University), Lower Egypt (Mansoura University and Zagazig University), and the Frontier region (Arish University and New Valley University). Public universities were selected to ensure representation of Egypt’s cultural diversity and to include a broader student demographic, while also accounting for the availability of local data collaborators.

### Study population and sampling technique

This study targeted undergraduate medical students from the first to the fifth year and interns from the universities mentioned above. Participants not affiliated with the target universities or who did not provide consent to complete the questionnaire were excluded. Participants were proportionally allocated across each region based on the number of registered students in the medical faculties of the public universities. Convenience sampling was used due to logistical constraints, particularly the inability to contact all medical students for random sampling. To reduce the potential bias, we ensured geographical diversity and proportional representation across regions and educational years.

### Sample size calculation

A pilot study was conducted on 31 students from different educational years. It revealed that the mean score of the Cancer Stigma Scale (CASS) was 1.74 ± 0.75 Standard deviation (SD). Using MedCalc Statistical Software version 14.8.1 (MedCalc Software bvba, Ostend, Belgium), a minimum required sample size of 2188 medical students was calculated at a 95% confidence level, a 5% margin of error, and a design effect of four to compensate for the convenience sampling. However, 2,576 medical students completed the questionnaire and were included in the analysis.

### Study tool

The English questionnaire consisted of two sections. The first included socio-demographic, educational data, and knowledge-related questions, including age, gender, university, educational year, residence, family income, the student’s personal experience with BC (including their personal and family history of BC), whether they encountered someone with BC, the main sources from which they obtained information about BC—using a multiple-response checkbox (which allow participants to select more than one option), whether they studied BC in their curriculum, and whether they believe there is sufficient awareness about BC in their community.

The second section included the adapted CASS, which was developed and validated by Marlow et al. in 2014 for the non-cancer patient population [21]. The CASS consists of 25 items measuring six stigma domains: 1- Severity (perceptions of cancer's impact), 2- Financial Discrimination (support for restricting patients' rights), 3- Personal Responsibility (blame attribution), 4- Awkwardness (discomfort interacting with patients), 5- Avoidance (distancing behaviors), and 6- Policy Opposition (resistance to support systems). Responses were rated using a six-point Likert scale (1 = strongly disagree, 6 = strongly agree) and reverse-scored for 5 items, so all scores were from 1 to 6; higher scores indicated higher levels of stigma. We calculated mean scores of each domain (sum of the domain items' scores divided by their number), and a total mean score for all items of the CASS.

After a public health expert reviewed the questionnaire, a pilot study was conducted at Mansoura University on 31 students to ensure content validity, clarity, and completion time. The reliability of the CASS was assessed using Cronbach’s alpha test, which measures the internal consistency of the items. The CASS score showed good internal validity (Cronbach’s alpha = 0.94), and its domain values ranged from 0.72 to 0.97. To adapt the CASS for BC stigma, the word “cancer” was replaced by “breast cancer” in each item, and the “not sure” option was removed, following a previous adaptation [22]. The pilot data were excluded from the analysis.

### Data collection

Data were collected using a Google Forms questionnaire. Collaborators distributed the questionnaire via the students’ social media groups (e.g., Facebook, WhatsApp, and Telegram groups) across the selected universities. The form included the study’s goals and objectives, anonymity assurance, and informed consent. To avoid duplicate responses, Google Forms settings were adjusted to accept one response per email (without collecting emails to ensure anonymity). Participants who did not provide consent were excluded.

### Statistical analysis

The data were analyzed using IBM Statistical Package for Social Science (SPSS) software version 29 (IBM Corp., Armonk, NY, USA). Categorical variables were summarized as frequencies and percentages (%). Continuous variables (CASS scores) were tested for normality and found to be non-parametric in distribution. Therefore, they were summarized by median (min–max). Inferential tests: Mann–Whitney U test and Kruskal–Wallis test were used to compare group medians. Following a significant result from the Kruskal–Wallis test, Dunn’s pairwise comparison was used to adjust for multiple comparisons. Spearman’s correlation coefficient (r) was used to assess bivariate correlations. Multiple linear regression analysis was performed to identify factors associated with BC stigma scores. Before correlation and regression analysis, the CASS score was log-transformed to meet the normality assumption required for linear regression results. A p-value of ≤ 0.05 was considered statistically significant.

## Results

### Descriptive statistics of the 25-item CASS

The total score was relatively low (with a mean of 2.37 ± 0.83) and a median = 2.16 (36% of the total possible score of 6), with notable variation observed across different stigma domains. Stigma was highest in Severity (M = 3.00) and Financial Discrimination (M = 2.67) domains. Specifically, 58.8% agreed that cancer devastates the lives of those it touches, and 49.1% supported policy reconsideration for patients.

In contrast, the Avoidance domain (M = 1.00) was the lowest, with only 14.3% of participants indicating they would avoid a person with cancer. Similarly, the Policy Opposition statements received endorsement by only 2.8–5% of the participants (M = 1.33) (Table 1).

**Table 1:** Descriptive statistics of the 25 items of the Cancer Stigma Scale (CASS) and total score

	Agree^1^ n(%)	Disagree^2^ n(%)	Median (Min–Max)
Awkwardness			2.40 (1.00–6.00)
®I would feel at ease around someone with cancer	2183 (84.7%)	393 (15.3%)	
®I would feel comfortable around someone with cancer	2179 (84.6%)	397 (15.4%)	
I would find it difficult being around someone with cancer	834 (32.4%)	1742 (67.6%)	
I would find it hard to talk to someone with cancer	677 (26.3%)	1899 (73.7%)	
I would feel embarrassed discussing cancer with someone who had it	1216 (47.2%)	1360 (52.8%)	
Severity			3.00 (1.0- 6.00)
Once you’ve had cancer, you can never be ‘normal’ again	945 (36.7%)	1631 (63.3%)	
Having cancer usually ruins a person’s career	1164 (45.2%)	1412 (54.8%)	
Getting cancer means having to mentally prepare oneself for death	720 (28.0%)	1856 (72.0%)	
Cancer usually ruins close personal relationships	1093 (42.4%)	1483 (57.6%)	
Cancer devastates the lives of those it touches	1514 (58.8%)	1062 (41.2%)	
Avoidance			1.00 (1.00–6.00)
If a colleague had cancer, I would try to avoid them	392 (15.2%)	2184 (84.8%)	
I would distance myself physically from someone with cancer	424 (16.5%)	2152 (83.5%)	
I would feel irritated by someone with cancer	421 (16.3%)	2155 (83.7%)	
I would try to avoid a person with cancer	368 (14.3%)	2208 (85.7%)	
I would feel angered by someone with cancer	379 (14.7%)	2197 (85.3%)	
Policy Opposition			1.33 (1.00–6.00)
®More government funding should be spent on the care and treatment of those with cancer	2477 (96.2%)	99 (3.8%)	
®The needs of people with cancer should be given top priority	2448 (95.0%)	128 (5.0%)	
®We have a responsibility to provide the best possible care for people with cancer	2503 (97.2%)	73 (2.8%)	
Responsibility			2.50 (1.00–6.00)
A person with cancer is liable for their condition	1465 (56.9%)	1111 (43.1%)	
A person with cancer is accountable for their condition	1087 (42.2%)	1489 (57.8%)	
If a person has cancer, it’s probably their fault	533 (20.7%)	2043 (79.3%)	
A person with cancer is to blame for their condition	479 (18.6%)	2097 (81.4%)	
Discrimination			2.67 (1.00–6.00)
It is acceptable for banks to refuse to make loans to people with cancer	516 (20.0%)	2060 (80.0%)	
Banks should be allowed to refuse mortgage applications for cancer-related reasons	656 (25.5%)	1920 (74.5%)	
It is acceptable for insurance companies to reconsider a policy if someone has cancer	1264 (49.1%)	1312 (50.9%)	
Total CASS Score			2.16 (1.00–5.80)

^1^Agree included: slightly agree, moderately agree, and strongly agree

^2^Disagree included: slightly disagree, moderately disagree, and strongly disagree

®Reversed items

### Variation of CASS scores by socio-demographics

Of the 2,576 participants, ages ranged from 17 to 31 Years with a mean of 21.33 ± 2.18 SD. Female accounted for 56.5%, 53.5% were aged 21 or younger, and participants were distributed evenly across the educational years. Most resided in urban areas (73.6%), and 71.0% reported having sufficient financial income. Only 2.3% of the participants were from the Frontier governorates (Table 2).

**Table 2:** Variation of total CASS score and its domains with socio-demographic characteristics of study participants (*N* = 2576)

Characteristic	Total n(%)	Total CASS score median (min- max)	Awkwardness median (min–max)	Severity median (min–max)	Avoidance median (min–max)	Policy Opposition median (min–max)	Responsibility median (min–max)	Discrimination median (min- max)
Sex								
Male	1456 (56.5%)	2.20 (1.00–4.94)	2.20 (1.00–5.60)	3.00 (1.00–6.00)	1.00 (1.00–6.00)	1.67 (1.00–6.00)	2.50 (1.00–6.00)	2.33 (1.00–6.00)
Female	1120 (43.5%)	2.12 (1.00–5.80)	2.40 (1.00–6.00)	2.80 (1.00–6.00)	1.00 (1.00–6.00)	1.33 (1.00–6.00)	2.25 (1.00–6.00)	2.67 (1.00–6.00)
* p*-value ^***x***^		**< 0.001**	**0.001**	**< 0.001**	**< 0.001**	**< 0.001**	**< 0.001**	0.575
Age group								
21 and younger	1379 (53.5%)	2.12 (1.00–5.16)	2.40 (1.00–6.00)	2.80 (1.00–6.00)	1.00 (1.00–6.00)	1.33 (1.00–6.00)	2.50 (1.00–6.00)	2.67 (1.00–6.00)
22 and older	1197 (46.5%)	2.20 (1.00–5.80)	2.40 (1.00–6.00)	3.20 (1.00–6.00)	1.00 (1.00–6.00)	1.33 (1.00–6.00)	2.50 (1.00–6.00)	2.67 (1.00–6.00)
* p*-value ^***x***^		**0.001**	0.155	**< 0.001**	0.163	0.553	**0.019**	0.751
Region								
Frontiers Governorates	60 (2.3%)	2.24 (1.20–4.48)	2.50 (1.00–4.60)	2.60 (1.00–5.20)	1.00 (1.00–5.20)	1.33 (1.00–4.33)	3.00 (1.00–5.50)	3.00 (1.00–5.67)
Lower Egypt Governorates	821 (31.9%)	2.20 (1.00–5.08) ^***a,b***^	2.40 (1.00–5.60)	2.80 (1.00–6.00)	1.00 (1.00–6.00)	1.33 (1.00–6.00)	2.50 (1.00–6.00)	2.67 (1.00–6.00)
Upper Egypt Governorates	466 (18.1%)	2.30 (1.00–4.64) ^***b,c***^	2.70 (1.00–5.20)	3.00 ^***a***^ (1.00–5.60)	1.00 (1.00–6.00)	1.33 (1.00–6.00)	2.75 (1.00–5.50)	2.67 (1.00–6.00)
Urban Governorates	1229 (47.7%)	2.08 (1.00–5.80) ^***a,c***^	2.20 (1.00–6.00)	3.00 (1.00–6.00)	1.00 (1.00–6.00)	1.33 (1.00–6.00)	2.25 (1.00–6.00)	2.33 (1.00–6.00)
* p*-value*		**< 0.001**	**< 0.001**	0.110	**0.001**	**< 0.001**	**< 0.001**	**< 0.001**
Educational year								
1 st year	387 (15%)	2.16 (1.04–5.16)^***e,f***^	2.40 (1.00–5.60)	2.80 (1.00–6.00)	1.00 (1.00–6.00)	1.33 (1.00–6.00)	2.50 (1.00–6.00)	2.67 (1.00–6.00)
2nd year	423 (16.4%)	2.12 (1.00–4.68) ^***a***^	2.20 (1.00–5.60)	3.00 (1.00–6.00)	1.00 (1.00–6.00)	1.33 (1.00–5.33)	2.25 (1.00–6.00)	2.67 (1.00–6.00)
3rd year	451 (17.5%)	2.12 (1.00–4.96) ^***b***^	2.40 (1.00–6.00)	2.60 (1.00–6.00)	1.00 (1.00–6.00)	1.33 (1.00–6.00)	2.50 (1.00–6.00)	2.67 (1.00–6.00)
4th year	456 (17.7%)	2.16 (1.08–5.80) ^***c,g***^	2.20 (1.00–6.00)	3.20 (1.00–6.00)	1.00 (1.00–6.00)	1.33 (1.00–6.00)	2.50 (1.00–6.00)	2.67 (1.00–6.00)
5th year	413 (16.0%)	2.28(1.08–4.80)^***a,b,d,f,g***^	2.60 (1.00–5.60)	3.20 (1.00–5.80)	1.00 (1.00–5.60)	1.33 (1.00–6.00)	2.50 (1.00–6.00)	2.67 (1.00–6.00)
Intern	446 (17.3%)	2.12 (1.00–4.92) ^***c,d,e***^	2.40 (1.00–5.20)	2.80 (1.00–6.00)	1.00 (1.00–6.00)	1.33 (1.00–5.67)	2.25 (1.00–6.00)	2.17 (1.00–6.00)
* p*-value*		**< 0.001**	0.182	**< 0.001**	**0.005**	0.053	**< 0.001**	**< 0.001**
Residence								
Rural	680 (26.4%)	2.24 (1.00–5.80)	2.60 (1.00–6.00)	3.00 (1.00–6.00)	1.00 (1.00–6.00)	1.33 (1.00–6.00)	2.50 (1.00–6.00)	2.67 (1.00–6.00)
Urban	1896 (73.6%)	2.12 (1.00–5.16)	2.20 (1.00–6.00)	2.80 (1.00–6.00)	1.00 (1.00–6.00)	1.33 (1.00–6.00)	2.50 (1.00–6.00)	2.67 (1.00–6.00)
* p*-value ^***x***^		**< 0.001**	**< 0.001**	**0.003**	**< 0.001**	0.127	**0.003**	**< 0.001**
Family income								
Less than or barely sufficient	421 (16.3%)	2.28 (1.16–5.80)^***a,b***^	2.40 (1.00–6.00)	3.20 (1.00–6.00)	1.00 (1.00–6.00)	1.33 (1.00–6.00)	2.75 (1.00–6.00)	2.67 (1.00–6.00)
Sufficient	1828 (71.0%)	2.16 (1.00–5.16) ^***a,c***^	2.40 (1.00–5.60)	2.80 (1.00–6.00)	1.00 (1.00–6.00)	1.33 (1.00–6.00)	2.50 (1.00–6.00)	2.67 (1.00–6.00)
More than sufficient	327 (12.7%)	2.00 (1.00–4.68)^***b,c***^	2.20 (1.00–5.60)	2.60 (1.00–6.00)	1.00 (1.00–6.00)	1.33 (1.00–6.00)	2.00 (1.00–5.75)	2.33 (1.00–6.00)
* p*-value*		**< 0.001**	**< 0.001**	**< 0.001**	**< 0.001**	0.053	**< 0.001**	**< 0.001**

^*^p-value was calculated using KW test (Kruskal–Wallis H test)

^x^*p*-value was calculated using Mann–Whitney U test

^a,b,c,d,e,f,g^are significant (*P*-value < 0.05) between the corresponding groups using Dunn’s pairwise comparison by Post-Hoc analysis, significant *p*-values (< 0.05) are bold

Male students had significantly higher total stigma scores than females (2.20 vs. 2.12; *p* < 0.001), particularly in Severity, Policy Opposition, Personal Responsibility, and Avoidance (all *p* ≤ 0.001), despite identical median values in some cases. Conversely, females reported significantly higher Awkwardness (median = 2.40 vs. 2.20; *p* = 0.001).

Students aged 22 or older had higher total stigma scores (2.20 vs. 2.12; *p* = 0.001), especially in the Severity domain (3.20 vs. 2.80; *p* < 0.001). Students from Upper Egypt exhibited the highest stigma score (2.30; *p* < 0.001), with significant differences across all domains. Fifth-year students exhibited higher stigma levels (2.28; *p* < 0.001), with notable variation in Severity, Avoidance, Personal Responsibility, and Financial Discrimination.

Rural students also had higher stigma scores (2.24 vs 2.12; *p* < 0.001), particularly in Awkwardness, Severity, and Financial Discrimination (all *p* ≤ 0.003). Participants from families with less than sufficient income had the highest score (2.28; *p* < 0.001), especially in Severity and Personal Responsibility (both *p* < 0.001).

### Variation of CASS by academic, clinical history, and knowledge-related variables

In Table 3, students with a personal history of breast problems reported higher stigma scores (2.40 vs. 2.16; *p* < 0.001), significantly higher across all major domains, including Awkwardness, Severity, Avoidance, Personal Responsibility, and Financial Discrimination. Interestingly, those who believed there was sufficient community awareness about BC reported a higher total stigma score (2.28 vs. 2.12; *p* < 0.001). Significant differences were found in Awkwardness, Severity, Financial Discrimination, and Personal Responsibility (Table 3).

**Table 3:** Variation of total CASS score and its domains with academic, clinical history, knowledge-related characteristics of study participants (*N* = 2576)

Characteristic	Total n (%)	Total CASS score median (min – max)	Awkwardness median (min–max)	Severity median (min–max)	Avoidance median (min–max)	Policy Opposition median (min–max)	Responsibility median (min–max)	Discrimination median (min- max)
Contact with breast cancer patient								
No	1451 (56.3%)	2.16 (1.00–5.16)	2.40 (1.00–5.60)	2.80 (1.00–6.00)	1.00 (1.00–6.00)	1.33 (1.00–6.00)	2.50 (1.00–6.00)	2.67 (1.00–6.00)
Yes	1125 (43.7%)	2.12 (1.00–5.80)	2.20 (1.00–6.00)	3.00 (1.00–6.00)	1.00 (1.00–6.00)	1.33 (1.00–6.00)	2.25 (1.00–6.00)	2.33 (1.00–6.00)
*p*-value^***x***^		0.219	**0.002**	**< 0.001**	**0.008**	**< 0.001**	**0.040**	**0.012**
Breast cancer in close friends/family members								
No	1825 (70.8%)	2.16 (1.00–5.16)	2.40 (1.00–6.00)	3.00 (1.00–6.00)	1.00 (1.00–6.00)	1.33 (1.00–6.00)	2.50 (1.00–6.00)	2.67 (1.00–6.00)
Yes	751 (29.2%)	2.12 (1.04–5.80)	2.40 (1.00–6.00)	2.80 (1.00–6.00)	1.00 (1.00–6.00)	1.33 (1.00–6.00)	2.25 (1.00–6.00)	2.67 (1.00–6.00)
* p*-value^***x***^		0.176	0.082	0.395	**0.035**	**< 0.001**	0.065	0.536
History of breast problems								
No	2374 (92.2%)	2.16 (1.00–5.16)	2.40 (1.00–6.00)	2.80 (1.00–6.00)	1.00 (1.00–6.00)	1.33 (1.00–6.00)	2.50 (1.00–6.00)	2.67 (1.00–6.00)
Yes	202 (7.8%)	2.40 (1.00–5.80)	3.00 (1.00–6.00)	3.20 (1.00–6.00)	1.20 (1.00–6.00)	1.33 (1.00–6.00)	2.75 (1.00–6.00)	3.00 (1.00–6.00)
* p*-value^***x***^		**< 0.001**	**< 0.001**	**< 0.001**	**< 0.001**	0.831	**0.011**	**< 0.001**
Family history of breast cancer								
No	2205 (85.6%)	2.16 (1.00–5.16)	2.40 (1.00–6.00)	3.00 (1.00–6.00)	1.00 (1.00–6.00)	1.33 (1.00–6.00)	2.50 (1.00–6.00)	2.67 (1.00–6.00)
Yes	371 (14.4%)	2.12 (1.04–5.80)	2.40 (1.00–6.00)	3.00 (1.00–6.00)	1.00 (1.00–6.00)	1.33 (1.00–6.00)	2.25 (1.00–6.00)	2.67 (1.00–6.00)
* p*-value^***x***^		0.290	0.132	0.959	**0.001**	**0.007**	0.078	0.504
Studied breast cancer in your curriculum								
No	794 (30.8%)	2.12 (1.00–4.96)	2.40 (1.00–5.60)	2.80 (1.00–6.00)	1.00 (1.00–6.00)	1.33 (1.00–6.00)	2.50 (1.00–6.00)	2.67 (1.00–6.00)
Yes	1782 (69.2%)	2.16 (1.00–5.80)	2.40 (1.00–6.00)	3.00 (1.00–6.00)	1.00 (1.00–6.00)	1.33 (1.00–6.00)	2.50 (1.00–6.00)	2.67 (1.00–6.00)
* p*-value^***x***^		0.473	0.831	0.444	**0.050**	0.690	0.268	0.720
Believe there is sufficient awareness about breast cancer in your community								
No	1842 (71.5%)	2.12 (1.00–5.80)	2.20 (1.00–6.00)	2.80 (1.00–6.00)	1.00 (1.00–6.00)	1.33 (1.00–6.00)	2.25 (1.00–6.00)	2.33 (1.00–6.00)
Yes	734 (28.5%)	2.28 (1.00–5.16)	2.40 (1.00–5.60)	3.00 (1.00–6.00)	1.00 (1.00–6.00)	1.33 (1.00–6.00)	2.75 (1.00–6.00)	2.67 (1.00–6.00)
* p*-value^***x***^		**< 0.001**	**0.009**	**0.025**	**< 0.001**	0.311	**< 0.001**	**< 0.001**
Breast cancer is the most common cancer in women								
I don’t know	385 (14.9%)	2.20 (1.12–4.96)	2.40 (1.00–5.60)	3.00 (1.00–6.00)	1.00 (1.00–6.00)	1.67 (1.00–5.33)	2.50 (1.00–6.00)	2.67 (1.00–6.00)
No	124 (4.8%)	2.16 (1.04–4.72)	2.40 (1.00–5.20)	2.80 (1.00–5.80)	1.00 (1.00–5.40)	1.33 (1.00–6.00)	2.50 (1.00–5.75)	2.67 (1.00–6.00)
Yes	2067 (80.2%)	2.16 (1.00–5.80)	2.40 (1.00–6.00)	3.00 (1.00–6.00)	1.00 (1.00–6.00)	1.33 (1.00–6.00)	2.50 (1.00–6.00)	2.67 (1.00–6.00)
* p*-value*		0.655	**0.039**	0.677	0.282	**0.004**	0.977	0.329
Believe that a person's lifestyle can be a cause of breast cancer								
No	524 (20.3%)	2.12 (1.00–5.16)	2.40 (1.00–5.60)	2.80 (1.00–6.00)	1.00 (1.00–6.00)	1.33 (1.00–6.00)	2.25 (1.00–6.00)	2.67 (1.00–6.00)
Yes	2052 (79.7%)	2.16 (1.00–5.80)	2.40 (1.00–6.00)	3.00 (1.00–6.00)	1.00 (1.00–6.00)	1.33 (1.00–6.00)	2.50 (1.00–6.00)	2.67 (1.00–6.00)
* p*-value^***x***^		0.553	**0.025**	0.674	0.942	0.503	**0.004**	0.846

^*^p-value was calculated using KW test (Kruskal–Wallis H test)

^x^*p*-value was calculated using Mann–Whitney U test

^a,b,c^are significant (*P*-value < 0.05) between the corresponding groups using Dunn’s pairwise comparison by Post-Hoc analysis, significant *p*-values (< 0.05) are bold

### Correlation and linear regression

Table 4 presents the correlation and linear regression analyses between the log-total CASS score and associated factors. A significant negative correlation was observed with sex (*r* = −0.1, *p* < 0.001), region (*r* = −0.1, *p* < 0.001), family income (*r* = −0.11, *p* < 0.001), history of breast problems (*r* = −0.08, *p* < 0.001), and sufficient awareness about BC (*r* = −0.1, p < 0.001). In contrast, a significant positive correlation was observed in residence (*r* = 0.09, *p* = 0.001) and age (*r* = 0.04, *p* = 0.03).

**Table 4:** Correlation and linear regression analysis of Cancer Stigma Scores

	Log total CASS score				
	Correlation		Linear regression		
	*r*	*P*	β	t	*P*
Sex	−0.1	< 0.001	−0.02	4.2	< 0.001
Age	0.04	0.03	0.01	4.2	< 0.001
Region	−0.1	< 0.001	−0.02	5.2	< 0.001
Educational year	−0.002	0.9	−0.01	3.7	< 0.001
Residence	0.09	0.001	0.02	3.2	< 0.001
Family income	−0.11	< 0.001	−0.025	4.8	< 0.001
Contact with breast cancer patient	0.02	0.22	0.006	1.0	0.3
Breast cancer in close friends/family members	0.03	0.2	0.003	0.4	0.7
Family history of breast cancer	0.02	0.3	0.003	0.3	0.7
History of breast problems	−0.08	< 0.001	−0.05	5.1	< 0.001
Studied breast cancer in the curriculum	−0.01	0.47	0.002	0.3	0.7
Sufficient awareness about breast cancer	−0.1	< 0.001	−0.04	6.3	< 0.001
Constant Model ANOVA R^2^			0.44 F = 14.3; *P* < 0.001 0.06		

*r* = Spearman coefficient, β = unstandardized coefficient. Categorical variables were included in the correlation and linear regression: sex coded as male = 1, female = 2. Regions were categorized as upper Egypt = 1, lower Egypt = 2, urban = 3, frontiers = 4. Residence classified as urban = 1, rural = 2. Family income is classified as less than sufficient = 1, sufficient = 2, and more than sufficient = 3. The academic, experimental, and knowledge-related questions as yes = 1 and no = 2

Several variables were significantly associated with the log-transformed CASS score in the multiple linear regression model. These included sex (β = −0.02, *p* < 0.001), age (β = 0.01, *p* < 0.001), region (β = −0.02, *p* < 0.001), educational year (β = −0.01, *p* < 0.001), residence (β = 0.02, *p* < 0.001), family income (β = −0.025, *p* = 0.001), personal history of breast problems (β = −0.05, *p* < 0.001), and perception of sufficient community awareness about BC (β = −0.04, *p* < 0.001) (Table 4).

### Main sources of information about breast cancer

**Fig. 1:**
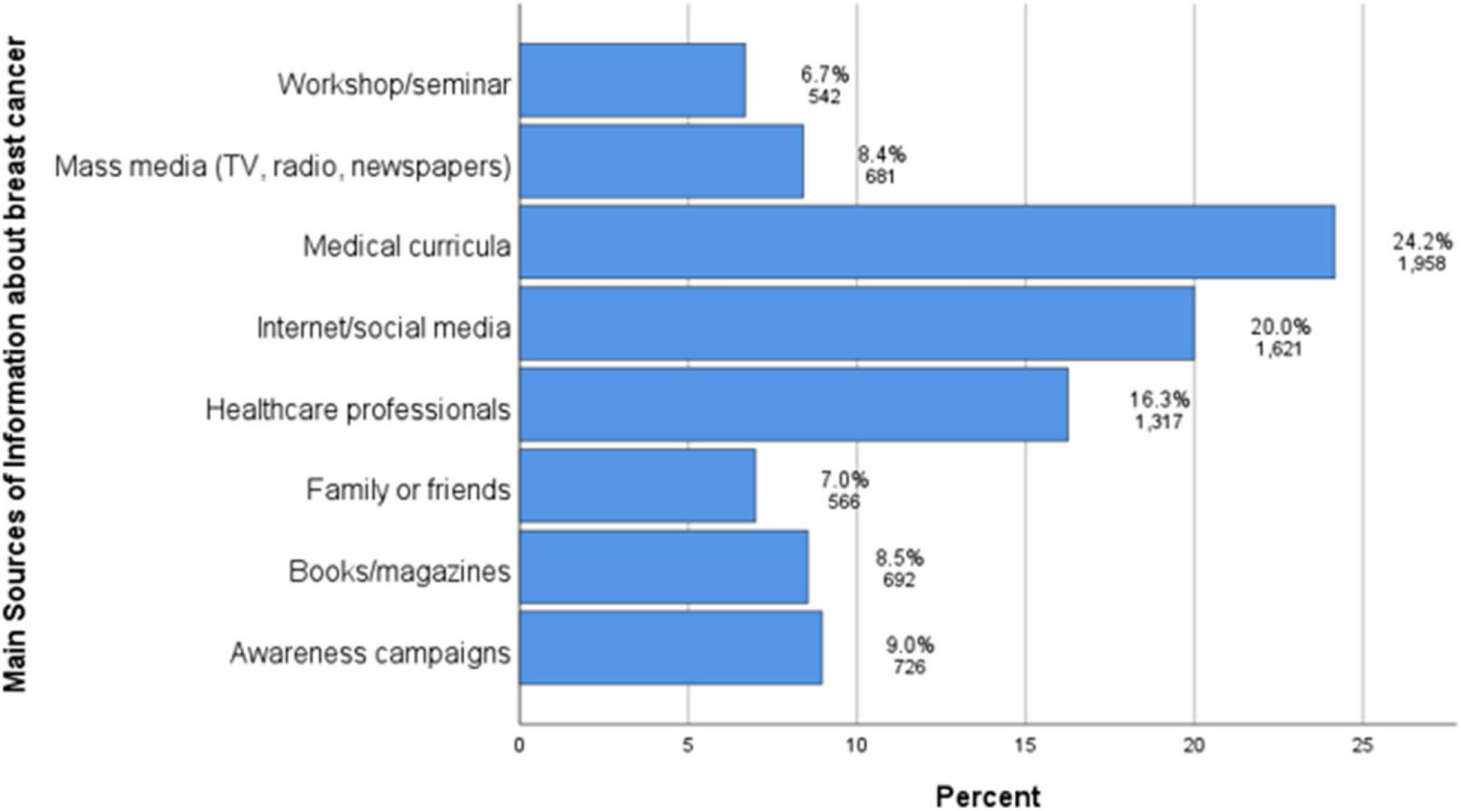
Main sources of information about breast cancer among medical students in Egypt (*N* = 2576). Note: categories are not mutually exclusive

Figure 1 shows the main sources of information about BC among participants. Medical curricula were reported as the primary source of their information (24.2%), followed by internet/social media (20.0%) and healthcare professionals (16.3%). In contrast, workshops/seminars (6.7%) and family or friends (7.0%) were the least dependent sources of information (Fig. 1).

## Discussion

As key figures within healthcare settings, physicians must be especially empathetic and aware of the impact of stigma, as the perceptions of these healthcare professionals directly influence clinical care and patients' health [17]. However, even though BC is widely regarded as a stigmatized topic given its close association with female identity and socially sensitive nature, both locally and globally [23–25], stigma among medical students, who represent future healthcare providers, has yet to be explored in existing research. Recognizing this gap, we used an adapted version of the CASS tool to evaluate the extent of medical students' stigma towards BC and identify its associated factors. To the best of our knowledge, this was the first study in Egypt and the world to employ the CASS among medical students, which could serve as a stepping-stone for evaluating BC stigma in the general population. We aimed to use our findings to pinpoint adjustments needed to the current medical education system, with the ultimate goal of supporting broader public health initiatives.

The observed low median CASS score (below 50% of the maximum 6 points) likely reflected medical students' robust oncology education, consistent with studies from Egypt [18], Saudi Arabia [26], and South India [27] that demonstrated superior cancer knowledge among medical trainees. However, even minimal stigma levels warrant clinical attention, as evidence confirms these can manifest through microaggressions or implicit biases that compromise care quality [28]. This highlights the need for early, structured training that goes beyond medical knowledge to include communication without judgment, active listening, and self-awareness. Supporting future healthcare professionals in developing these skills helps create a care environment where patients feel truly heard, respected, and safe — and that can make all the difference. Yet, some domains of stigma were more pronounced than others. Higher stigma levels were found in the Severity domain, followed by Financial Discrimination and Personal Responsibility. Meanwhile, respondents were less stigmatizing of the parts on Policy Opposition and Avoidance. Having Severity as the domain with the highest score and Avoidance with the lowest was precisely in line with the results of two extensive general population studies conducted in Saudi Arabia [29] and England [22], as well as a study involving university students in Malaysia [30]. Participants' elevated Severity domain scores reflected both legitimate concerns about breast cancer outcomes in Egypt and potential misinformation exposure. Egypt's documented breast cancer mortality rate of 53.5% significantly exceeded global averages due to frequent late-stage diagnosis (71% of cases) and limited screening access (14% coverage) [31], objectively justifying heightened severity perceptions. Among the 20.0% of students relying primarily on social media for health information in our study, Chung et al. demonstrated that this exposure can directly increase cancer fatalism odds ratio (OR) = 2.3, 95% confidence interval (CI):1.8–3.1 through disproportionate representation of poor outcomes and minimal survivorship narratives [32]. Medical curricula should counter this by: incorporating critical evaluation of online health content, featuring survivor testimonials, and contextualizing Egyptian outcomes alongside global survival data to demonstrate treatability. These educational reforms should accompany the expansion of the national screening program to align perceived and actual disease trajectories [31, 33]. Achieving this also required equipping both the public and future healthcare providers with clear, evidence-based information about cancer outcomes and proactively addressing fatalistic beliefs through structured education and communication.

In contrast, medical students in our sample demonstrated the least stigmatizing attitudes in Avoidance, when compared to the rest of the domains. Physical changes brought on by cancer treatment, such as alopecia and surgical scars, were thought to have an impact on cancer patients' relationships and social interactions, making them feel rejected and excluded [34]. Particularly in BC, where mastectomies are one of its core lines of treatment, some cultures regard them as mutilating [35]. In Middle Eastern societies, misconceptions that BC is contagious further contribute to social avoidance of those affected [9]. However, through their education in cancer biology, etiology, and treatment, medical students have come to understand the fact that cancer can only be transmitted genetically, and is therefore non-contagious in nature. Their familiarity with BC treatments and their outcomes also meant they are less likely to view interventions such as mastectomy as disfiguring or mutilating. As a result, medical students were less inclined to avoid those with BC and came to see them as 'normal' in contrast to the general population. Knowledge, however, can sometimes be a double-edged sword. Research has indicated that an awareness of the modifiable risk factors for cancer, such as smoking, obesity, poor eating habits, and physical inactivity, is associated with higher personal responsibility scores [36]. For medical students who had studied the pathophysiology and potential etiologies of various cancers, including BC, this is an evident fact. This was supported by our findings, since almost 80% of our sample of medical students reported that they believed a person’s lifestyle could be a cause of BC, with the bivariate analysis revealing a significant increase in their Personal Responsibility domain compared to those who do not hold the same belief. While it is important to raise awareness about the causes of cancer, it may unintentionally perpetuate the notion that cancer is self-inflicted and increase stigma towards patients. Special attention should be paid to medical curricula and online platforms, as these served as the primary sources of BC information for nearly half of the students in our sample. Therefore, medical curricula must strategically convey information in a manner that promotes awareness of risk factors whilst minimizing the potential for stigmatization [22]. For instance, it must be emphasized that cancer is a complex, multifactorial disease with a combination of genetic, environmental, and lifestyle elements collectively influencing its pathogenesis [37]. Medical curricula should frame discussions of modifiable risk factors by emphasizing their probabilistic nature rather than deterministic outcomes, while acknowledging that many patients face unavoidable risks as prior radiation therapy or genetic predisposition. Training should focus on teaching students to communicate risk information empathetically without implying blame, using techniques like patient-centered counseling that highlight structural barriers to healthy behaviors. Regular competency assessments through clinical simulations can help ensure graduates can discuss risk factors in ways that promote awareness without fostering stigma, ultimately improving patient trust and outcomes.

Meanwhile, medical students also demonstrated very low values of Policy Opposition towards BC. This could be explained by their awareness of the lengthy and costly nature of cancer treatment, making them more empathetic and supportive of resource allocation towards cancer care [38]. In addition, their sense of responsibility as future healthcare providers to act in the best interest of the patient and their commitment to improving health outcomes could have played a role. This, in turn, accentuates the importance of medical students’ involvement as policy advocates and even future policy-makers. Their engagement can help push for policies that address critical issues like BC treatment [39], aligning with the broader goals of the World Health Organization (WHO) Global Breast Cancer Initiative [40], as well as the United Nations (UN) 2030 Agenda for Sustainable Development [41]. A prominent example of organizations that encourage medical student advocacy and participation in health-related decision-making is the International Federation of Medical Students’ Associations (IFMSA). As one of the oldest and largest global bodies representing medical students, IFMSA provides a platform for students to contribute to policy development aimed at reducing health inequities and enhancing patient care worldwide [42].

In light of our data, men had higher stigma levels than women. This could be potentially due to women’s reported higher empathy levels [43] and greater involvement in cancer awareness campaigns, which often highlight topics like women's health and BC screening [44, 45]. This is consistent with the results of numerous studies, including ones conducted among university students in Malaysia [30], as well as the general population in England [22], Saudi Arabia [29], and Oman [46]. In contrast, one study amongst the general population in China found no association between gender and stigma levels [47]. However, this might have been due to their small sample size, as well as gender imbalance with an overrepresentation of female participants.

Interestingly, when it came to age, our findings showed that as individuals got older, their stigma levels got higher, especially within the severity domain. This closely matches the results of a Saudi Arabian study where increasing age was associated with greater stigma [29]. One possible explanation is that older students may have had past life experiences witnessing family or community members suffer or die from BC, thus causing them to retain a stronger emotional association of cancer with fatality or suffering, even after learning the clinical facts. At the same time, stigma varied across educational years, with fifth-year students exhibiting the highest levels of stigmatizing attitudes, specifically when it came to awkwardness and severity, while interns managed to rank among the lowest. The reason for heightened severity among fifth-years could be more rooted in their personal traditional views or cultural beliefs, reinforcing negative stereotypes. Furthermore, some students may have felt ill-prepared to sensitively engage with the social and emotional aspects of BC stigma, leading to increased feelings of uncertainty and awkwardness—particularly if they have not yet received adequate training in areas such as psycho-oncology or the psychological impact of stigma, which many medical programs do not dive into until later years [48]. Similarly, first-year students also demonstrated relatively high levels of stigma. The disparity between first-year students and interns likely stemmed from the intensive training and consecutive increased awareness and knowledge that interns receive in their final years, in contrast to first-year students, who naturally have limited experience and patient exposure, as they have only recently begun their medical journey [49]. These results underscored the pivotal role of advanced medical education and experiential learning in mitigating negative attitudes toward individuals with BC.

Our study on students indicated that those residing in rural regions, such as Upper Egypt, and those having "less than sufficient" income both exhibited significantly higher levels of BC stigma. This may be because Egypt, like other LMICs, serves as an example for nations facing rural–urban development gaps. According to a 2024 report by the UN's Economic and Social Commission for Western Asia (ESCWA), 28% of rural Egyptians are multidimensionally poor, more than twice the rate in urban areas (11.9%), due to a lack of services, housing, education, and employment opportunities [50]. Supporting this, the World Bank’s 2019 report, Understanding Poverty and Inequality in Egypt, highlights that most of the poor and vulnerable populations reside in the governorates of Upper Egypt, which are predominantly rural [51]. Higher levels of stigma observed among individuals with lower income may be associated with the increased financial challenges often faced by low-income households. The already high cost of cancer care could have contributed to BC patients being perceived as an additional economic burden, potentially reinforcing stigmatizing attitudes toward the disease [52, 53]. Additionally, localized cultural taboos, gender norms, and other social factors, which are more prominent in rural areas, may have influenced these perceptions [7, 24, 54]. Additionally, localized cultural taboos, gender norms, and other social factors, which are more prominent in rural areas, likely influenced these perceptions [7, 24, 54]. Resolving these issues requires two parallel strategies: first, community-based programs and awareness campaigns should be tailored to address the specific cultural and social dynamics of these regions; second, increased investment in healthcare infrastructure and access is critical, as greater accessibility can help normalize service utilization and reduce associated fear and shame. Together, these interventions can effectively tackle stigma at both the personal and community levels.

While contact with BC patients helped reduce feelings of Awkwardness, Personal Responsibility, and Financial Discrimination, our findings showed that it had no discernible impact on other stigma beliefs, such as perceived severity. Likewise, a large-scale Saudi Arabian study also reported persistent stigma despite patient contact [29]. The persistence of perceptions surrounding BC’s severity and life-threatening nature may stem from deeply ingrained cultural beliefs, exposure to inaccurate media portrayals, or personal experiences with advanced or fatal cases. Such beliefs may remain unchanged even after direct patient contact, particularly if the interactions are brief, emotionally distant, or involve visibly unwell patients. Initial or surface-level contact may not provide enough depth or emotional engagement to challenge internalized assumptions about the disease's severity meaningfully. This suggests that clinical exposure alone may not be enough to tackle all facets of stigma. However, a study at the Egyptian University of Port Said addressed this challenge by demonstrating that combining exposure with structured education led to more positive attitude changes [55], highlighting the potential value of integrating educational interventions to strengthen the effect of clinical contact. Similarly, our results also showed that having close friends or relatives with BC did not significantly alter perceptions of the disease's severity or personal responsibility. Given that medical students represent future healthcare providers, their attitudes are particularly important, as patients are likely to expect empathy and support, rather than stigma, from those in clinical roles.

Contrary to expectations, students with a personal history of breast-related conditions had higher stigma scores, particularly in terms of Awkwardness, Severity, and Financial Discrimination. While one might anticipate that personal experience would foster greater empathy and reduce stigma [56], the opposite was found. Individuals who have experienced breast health issues may have faced stigma themselves. This could have led them to internalize negative perceptions, which in turn affected their attitudes toward others with similar conditions [57]. This shows the importance of addressing emotional responses as part of increasing awareness within medical education.

### Strengths and limitations

Our study has several strengths and limitations. While the cross-sectional design offers valuable insights, it does not establish a definitive cause-and-effect relationship between stigma and its potential contributing factors. Longitudinal studies are encouraged to uncover these dynamics. Convenience sampling was adopted due to logistical constraints, including the reliance on available local collaborators across the multicenter universities. This method may have introduced a potential for selection bias, as participants were self-selected or recruited based on accessibility. However, to improve representativeness, the sample was proportionally allocated among medical students from different educational years across Egypt’s four main regions. Future studies should aim to include non-medical students and expand across multiple countries to gain broader insight into BC stigma.

Furthermore, this study employed a self-administered online questionnaire, which inevitably introduced the possibility of response bias, where participants may have misinterpreted the questions, leading to inaccurate answers. While stigma is generally perceived as socially undesirable, potentially influencing participants to respond more favorably, the anonymity of online questionnaires should likely minimize the impact of social desirability bias. Regarding our regression model, it explained 6% of the variation in the cancer stigma score, suggesting that additional unmeasured factors may contribute. Variables such as personal demographics (marital status, race, and religion), healthcare access, and media exposure patterns may play a role in shaping BC stigma. This limits the predictive utility of our identified variables, warranting further research to explore their effect. Additionally, while log transformation was necessary to address non-normality in our data, it is important to recognize that such transformations can alter the interpretation of effect sizes and potentially distort original data relationships. These analytical decisions should be considered when interpreting the regression results. Despite these limitations, this study provides initial evidence that BC stigma exists among future healthcare leaders, laying the groundwork for incorporating anti-stigma training into medical education and guiding public health initiatives to address existing gaps.

## Conclusion

BC stigma among medical students was relatively low, though some aspects were more prominent than others. The extent of BC stigma was associated with participants’ sociodemographic characteristics such as sex, age, educational year, residence, and income, as well as other factors like contact with BC patients and personal history of breast problems. Given their future roles as healthcare providers, medical students should be equipped with both the empathy and knowledge necessary to combat stigma within healthcare settings. Targeted interventions should involve curriculum reforms that integrate anti-stigma training, taking into account high-risk groups such as males and rural residents. Future research should expand to include non-medical student populations and adopt longitudinal designs to evaluate the effectiveness of stigma-reduction strategies. Broader cross-cultural comparisons may also reveal how different educational and healthcare systems mediate stigma, offering more globalized solutions to address this critical public health concern in LMICs.

## Supplementary Information

Supplementary material 1.

## Data Availability

The data of this study are available from the corresponding author upon reasonable request.

## Abbreviations

BC: Breast cancer
CASS: Cancer stigma scale
CI: Confidence interval
EWHI: Egyptian women’s health initiative
ESCWA: Economic and social commission for Western Asia
GLOBOCAN: Global cancer observatory
IFMSA: International federation of medical students’ associations
LMICs: Low- and middle-income countries
OR: Odds ratio
SD: Standard deviation
UN: United Nations
WHO: World health organization
WHOP: Women’s health outreach program
