# Supplementary material for: Exploring breast cancer stigma among medical students in Egypt: a national multi-center cross-sectional study
Source: BMC Public Health. 2025 Oct 27;25:3604. doi: 10.1186/s12889-025-24656-2 (PMC12557924; doi:10.1186/s12889-025-24656-2)
Supplement: Supplementary file 1 — Supplementary material 1. [file 12889_2025_24656_MOESM1_ESM.docx]

**Exploring breast cancer stigma among medical students in Egypt: A national multi-center cross-sectional study.**

This questionnaire aims to assess breast cancer stigma among medical students in Egypt. It is composed of**40 questions**and shouldn't take more than 5 minutes to finish. Your response would be appreciated!

**Please note that: your answers and information would only be accessed by the research team, used exclusively for research purposes, and completely secured.**

**Informed consent:**

- I voluntarily agree to participate in this research study.
- I understand that I will not benefit directly from participating in this study.
- I understand that participation involves answering the following questions, which will be processed and analyzed only by the researchers on this team.
- I understand that even if I agree to participate now, I can withdraw at any time or refuse to answer any question without any consequences of any kind.
- I understand that all information I provide for this study will be treated confidentially.

**Do you agree to the above terms?**

By clicking "Yes, I agree" you consent that you are willing to answer the questions in this survey.

Yes , I agree

No, I don`t agree

1. **Socio-demographic characteristics and clinical history:**

This section will help us assess the relation between **sociodemographic factors**, **breast cancer stigma level,** and **clinical history**.

**Sex:**

Male/ Female

**Age:**

**University:**

**Educational year:**

**Residence:** Urban / Rural

**Family income:** Less than or barely sufficient/ Barely sufficient/ Sufficient/ More than sufficient

**Have you ever been in contact with a breast cancer patient?**

Yes

No

**Do/did any of your close friends or family members have/had breast cancer?**

Yes

No

**Have you ever had any breast problems?**

Yes

No

**Do you have a family history of breast cancer?**

Yes

No

**What are the main sources from which you obtain information about breast cancer? (Multiple answers can be chosen)**

Healthcare professionals

Medical curricula

Workshop/seminar

Books/magazines

Mass media

Internet/social media

Family or friends

Awareness campaigns

**Have you studied breast cancer in your curriculum?**

Yes

No

**Do you believe there is sufficient awareness about breast cancer in your community?**

Yes

No

**Breast cancer is the most common cancer in women**

Yes

No

I don't know

**Do you believe that a person's lifestyle can be a cause of breast cancer?**

Yes

No

1. **Perception of breast cancer using CASS**

This section will help us evaluate how medical students perceive **breast cancer**and assess the **difference in stigma levels** between them.

1. **Once you’ve had breast cancer you’re never ‘normal’ again**

Strongly Disagree Moderately Disagree Slightly Disagree

Slightly Agree Moderately Agree Strongly Agree

1. **Getting breast cancer means having to mentally prepare oneself for death**

Strongly Disagree Moderately Disagree Slightly Disagree

Slightly Agree Moderately Agree Strongly Agree

1. **A person with breast cancer is to blame for their condition**

Strongly Disagree Moderately Disagree Slightly Disagree

Slightly Agree Moderately Agree Strongly Agree

1. **Having breast cancer usually ruins a person’s career**

Strongly Disagree Moderately Disagree Slightly Disagree

Slightly Agree Moderately Agree Strongly Agree

1. **A person with breast cancer is accountable for their condition**

Strongly Disagree Moderately Disagree Slightly Disagree

Slightly Agree Moderately Agree Strongly Agree

1. **Breast cancer usually ruins close personal relationships**

Strongly Disagree Moderately Disagree Slightly Disagree

Slightly Agree Moderately Agree Strongly Agree

1. **Breast cancer devastates the lives of those it touches**

Strongly Disagree Moderately Disagree Slightly Disagree

Slightly Agree Moderately Agree Strongly Agree

1. **A person with breast cancer is liable for their condition**

Strongly Disagree Moderately Disagree Slightly Disagree

Slightly Agree Moderately Agree Strongly Agree

1. **If a person has breast cancer it’s probably their fault**

Strongly Disagree Moderately Disagree Slightly Disagree

Slightly Agree Moderately Agree Strongly Agree

**10) I would feel at ease around someone with breast cancer**

Strongly Disagree Moderately Disagree Slightly Disagree

Slightly Agree Moderately Agree Strongly Agree

**11) I would feel comfortable around someone with breast cancer**

Strongly Disagree Moderately Disagree Slightly Disagree

Slightly Agree Moderately Agree Strongly Agree

**12) I would try to avoid a person with breast cancer**

Strongly Disagree Moderately Disagree Slightly Disagree

Slightly Agree Moderately Agree Strongly Agree

**13) I would feel angered by someone with breast cancer**

Strongly Disagree Moderately Disagree Slightly Disagree

Slightly Agree Moderately Agree Strongly Agree

**14) I would find it difficult being around someone with breast cancer**

Strongly Disagree Moderately Disagree Slightly Disagree

Slightly Agree Moderately Agree Strongly Agree

**15) I would find it hard to talk to someone with breast cancer**

Strongly Disagree Moderately Disagree Slightly Disagree

Slightly Agree Moderately Agree Strongly Agree

**16) I would feel irritated by someone with breast cancer**

Strongly Disagree Moderately Disagree Slightly Disagree

Slightly Agree Moderately Agree Strongly Agree

**17) I would feel embarrassed discussing breast cancer with someone who had it**

Strongly Disagree Moderately Disagree Slightly Disagree

Slightly Agree Moderately Agree Strongly Agree

**18) I would distance myself physically from someone with breast cancer**

Strongly Disagree Moderately Disagree Slightly Disagree

Slightly Agree Moderately Agree Strongly Agree

**19) If a colleague had breast cancer, I would try to avoid them**

Strongly Disagree Moderately Disagree Slightly Disagree

Slightly Agree Moderately Agree Strongly Agree

**20) It is acceptable for banks to refuse to make loans to people with breast cancer**

Strongly Disagree Moderately Disagree Slightly Disagree

Slightly Agree Moderately Agree Strongly Agree

**21) The needs of people with breast cancer should be given top priority**

Strongly Disagree Moderately Disagree Slightly Disagree

Slightly Agree Moderately Agree Strongly Agree

**22) More government funding should be spent on the care and treatment of those with breast cancer**

Strongly Disagree Moderately Disagree Slightly Disagree

Slightly Agree Moderately Agree Strongly Agree

**23) We have a responsibility to provide the best possible care for people with breast cancer**

Strongly Disagree Moderately Disagree Slightly Disagree

Slightly Agree Moderately Agree Strongly Agree

**24) Banks should be allowed to refuse mortgage applications**

**for breast cancer-related reasons**

Strongly Disagree Moderately Disagree Slightly Disagree

Slightly Agree Moderately Agree Strongly Agree

**25) It is acceptable for insurance companies to reconsider a policy if someone has breast cancer**

Strongly Disagree Moderately Disagree Slightly Disagree

Slightly Agree Moderately Agree Strongly Agree
